# PD227 Qualitative Assessment Of The Value Of The Magnetic Resonance Linear Accelerator To Guide Decisions On Its Optimal Use

**DOI:** 10.1017/S026646232400446X

**Published:** 2025-01-07

**Authors:** Marike Ulehake, Ellen Brunenberg, Marcia Tummers, Marcel Verheij, Janneke Grutters

## Abstract

**Introduction:**

The added value of medical technology is typically assessed in specific patient groups, which is less useful to guide decisions on optimal use. The magnetic resonance linear accelerator (MR-Linac) is a costly innovation that can enhance radiotherapy precision through daily imaging and plan adaptation. This study aimed to better understand its potential value and inform users on its optimal application.

**Methods:**

Semi-structured interviews (n=24) were conducted with stakeholders, ranging from end users (radiation oncologist, clinical physicists, radiotherapy technologists, managers) to patient representatives and policy officers (e.g., procurement, health insurance, health technology assessment agency, and regulatory body). Themes explored with end users focused on the decision-making process of acquisition, current use, potential future deployment, optimal use, and the added value of MR-Linac. Themes explored with policymakers and patient representatives included their roles concerning costly medical technologies and their perspectives on the added value of the MR-Linac. Interview data were analyzed using thematic analysis.

**Results:**

Preliminary results showed stakeholders’ optimism for improved patient outcomes, including reduced toxicity and increased treatment efficacy. Patient representatives highlighted not only the clinical value, but also the importance of ensuring patients’ access to innovative care. End users perceived the MR-Linac as a gradual improvement, placing trust in its theoretical benefits. Additionally, they found value in its contribution to providing innovative treatment, potentially benefiting hospitals by attracting new patients. They also experienced improved job satisfaction. Policy officers emphasized the need for evidence to support potential clinical advantages as well as the significant costs associated with the MR-Linac within the broader healthcare context.

**Conclusions:**

This qualitative, technology focused study deepens our understanding of the potential added value of the MR-Linac, beyond a specific context or patient group. The results not only highlight potential patient benefits, but also show a broader significance of the MR-Linac at other levels (e.g., clinician, hospital, and society). It is important to consider these different levels when guiding decisions on use of the MR-Linac.

